# Advancing tuberculosis elimination in India: A qualitative review of current strategies and areas for improvement in tuberculosis preventive treatment

**DOI:** 10.1016/j.ijregi.2024.100556

**Published:** 2024-12-21

**Authors:** Harsh Shah, Jay Patel, Sandeep Rai, Abhishek Sen

**Affiliations:** Department of Public Health Science, Indian Institute of Public Health Gandhinagar (IIPHG), Gandhinagar, India

**Keywords:** Tuberculosis, Tuberculosis preventive treatment, National Tuberculosis Elimination Programme, Diagnostics, TB elimination, Latent TB infection

## Abstract

Tuberculosis (TB), caused by *Mycobacterium tuberculosis*, remains a global health challenge, despite being preventable and curable. The global goal to end TB by 2030, with India targeting a 90% reduction in TB incidence by 2025, demands comprehensive interventions in prevention, early detection, treatment, and capacity building. This study analyzes the 2023 Global TB Reports, published articles, guidelines, and data to explore India's current TB landscape. Despite progress in diagnostics and TB preventive treatment, significant challenges persist in accessibility, adherence, and treatment completion. Although advancements in household contact screening and TB preventive treatment initiation are notable, gaps in reporting, diagnostic algorithms, and adherence remain. Innovative diagnostic tools and shorter treatment regimens show promise but require more comprehensive implementation. India's strategy for TB elimination focuses on policy expansion, community engagement, and system enhancements. Continuous innovation, rigorous evaluation, and collaborative efforts are crucial to overcoming challenges and meeting TB elimination targets.

## Highlights

• Tuberculosis (TB) preventive treatment (TPT) is vital for India's TB elimination by 2025.
• Diagnostic innovations and shorter TPT regimens improve TB prevention efforts.
• Gaps persist in TPT adherence and reporting, despite increased screening.
• India's strategic TB approach focuses on policy expansion and system strengthening.
• Community engagement and innovations are critical to overcoming TB challenges.

## Introduction

Tuberculosis (TB) is an infectious disease caused by the *Mycobacterium tuberculosis* (MTB) and stands as a significant contributor to global morbidity and mortality. Affecting predominantly adults, with a higher prevalence among men, TB remains a persistent public health challenge [1]. Despite being curable and preventable, the disease has endured for centuries. The global commitment to end the TB epidemic by 2030 has set targets, with the international community aiming for a 90% reduction in TB deaths and an 80% reduction in the TB incidence rate by 2030 compared with 2015 [2]. In pursuit of this goal, India has adopted an even more ambitious target, aspiring to reduce TB incidence by 90% by 2025 [3]. Achieving an annual reduction of 10% in TB incidence is crucial to realizing this target. This necessitates comprehensive interventions across multiple pillars, including prevention, early detection, effective treatment, and capacity building [4,5].

The World Health Organization (WHO) advises offering TB preventive treatment (TPT) to household contacts (HHC) with TB infection (TBI) after clearing out active disease to lower the risk of TB disease among them. Pillar one of the WHO End TB Strategy also relies substantially on TPT, with a global goal of 90% TPT coverage among People living with HIV (PLHIV) and household contacts of people with pulmonary TB by 2025 [6]. For a variety of target groups, TPT successfully lowers the risk of illness and death. The access and uptake of TPT among individuals at risk is still poor, despite global commitment and efforts [7].

The primary objective of this review study is to offer qualitative insights into the current landscape of diagnostic options and TPT in India. To achieve this aim, an extensive analysis of published scholarly articles, guidelines, reports, and data extracted from the Global TB Reports of 2023 was undertaken. Through this qualitative exploration, the study aimed to provide a comprehensive understanding of the prevailing scenario and ongoing efforts and to identify areas for improvement that can contribute to India's ambitious goal of TB elimination, as outlined in the National Tuberculosis Elimination Programme (NTEP).

## Understanding tuberculosis infection & TPT

TBI is characterized as a persistent immune response to MTB antigens without clinical evidence of active TB [8]. The significance of TBI as a public health concern was underscored in 2015 with the introduction of the End TB Strategy and the release of initial guidelines on TBI management by the WHO [2]. This marked the first explicit national-level guidance on the programmatic management of TBI, outlining strategic actions to align with the goals of the End TB Strategy [4]. The purpose of TPT is to prevent the progression to active TB, acting against “dormant” bacilli within granulomas.

The strategic approach to TB elimination emphasizes two key actions. First, the rapid diagnosis and effective treatment of every individual developing active TB, estimated at 10 million cases annually, are paramount. This not only benefits the individual but also mitigates the risk of further MTB transmission within families, close contacts, and the broader community [9]. Second, among the estimated 1.7 billion individuals worldwide with latent MTB infection, identifying and treating those at risk of progressing to active disease is imperative [10].

According to the WHO, the regimens for TPT are categorized into three categories: isoniazid monotherapy (6 or 9 months), rifamycin-based shorter treatments, and levofloxacin for individuals exposed to multi-drug–resistant or rifampicin-resistant TB (RR-TB). Isoniazid preventive treatment has historically been the primary method for TPT, showing a significant reduction in TB risk, especially among individuals with HIV. However, recent evidence supports the efficacy of shorter rifamycin-based regimens, such as 3 months of weekly isoniazid and rifapentine (3HP) and daily rifapentine plus isoniazid for 1 month, which offer better adherence and fewer side effects than traditional isoniazid preventive treatment.

The WHO has expanded its recommendations to include various regimens suitable for all TB burden settings, provided that active TB disease can be reliably ruled out before starting treatment. In addition to the established options, the 2024 guidelines introduced levofloxacin (6Lfx) as a preventive option for those exposed to multi-drug–resistant/rifampicin-resistant TB. These updates aim to enhance accessibility and effectiveness of TPT worldwide, particularly among high-risk populations, while ensuring that cost does not hinder the provision of these essential treatments [8].

## Burden of TB infection

In terms of the latent TB burden, approximately a quarter of the world's population is estimated to have latent TB worldwide. A minor fraction of individuals with a TBI advance to complete TB disease, with an estimated annual incidence of 2.6 million cases [11]. Of these individuals with TBI, around 5% develop active disease within 2 years [12]. The National TB Prevalence Survey in India for the period 2019-2021 reported a TBI prevalence of 21.7% in individuals aged 15 years and older, using a robust standard error model [13]. A regional study conducted by Arohi *et al.* and Mohan *et al.* corroborated this finding and revealed that pooled prevalence could be 35-40%, indicating a comparable prevalence among the general population [14]. Also, few studies reported the risk of active TB after infection is several fold higher than traditionally accepted estimates and observed to be particularly high immediately after infection and in children, with high susceptibility among PLHIV and individuals with weakened immune systems [15].

## Management of TB infection

Although the early diagnosis and treatment of active TB remain key priorities in India, the prevention of TB is increasingly emphasized through the identification and treatment of TBI and active case finding in high-risk groups. TPT plays a critical role in reducing the risk of disease progression, with studies showing a 60% reduction in TB development and up to 90% among PLHIV [16]. However, approaches such as TPT alone may not suffice to achieve TB elimination. The overall infection rate of TB can only be reduced through a combination of interventions that address medical, social, and structural determinants of the disease.

In recognition of this, the Government of India has launched several initiatives, including the Pradhan Mantri TB Mukt Bharat Abhiyaan, which integrates nutritional and vocational support to improve treatment adherence and outcomes. Other efforts include the Programmatic Management of TPT, Programmatic Management of Drug-Resistant TB, community-led initiatives such as TB Mukt Villages, and the involvement of family caregivers for persons with TB. Such holistic approaches, which combine medical care with nutrition, social protection, and community engagement, are crucial for addressing the multifaceted challenges of TB and progressing toward India's ambitious 2025 elimination targets.

## Dimensions of TB infection diagnosis

### Screening and diagnosis of TB infection

Individuals at a heightened risk of MTB exposure and the progression from TBI to active TB significantly benefit from TBI screening [17]. In line with the WHO's post-2015 End TB Strategies recommendation for higher/upper-middle–income countries with a TB incidence of less than 100 per 100,000 individuals, systematic testing and treatment for TBI are strongly recommended [2]. However, conducting routine testing beyond these high-risk areas not only depletes valuable resources but also elevates the risk of encountering false-positive test results. The targeted Mantoux tuberculin skin test (TST) stands out as the widely used tool for TBI screening. The effect of Bacille Calmette-Guérin (BCG) vaccination on TST specificity varies based on the vaccine strain, age at vaccination, and dosage. Administered at birth in many regions, the BCG vaccine's impact on TST specificity is inconsistent [18].

As the global disease burden continues to escalate, there is a pressing need for improved TBI diagnostics options to halt and reverse this trend. Achieving efficient management of prophylactic treatments requires a sensitive and precise TBI diagnosis [19]. Although bacterial culture remains the gold standard for diagnosing active infection, there is no equivalent standard for detecting TBI. The diagnosis of TBI typically involves either an interferon-γ release assay (IGRA) or a TST [20]. In higher-middle– and high-income countries, the WHO recommends either IGRA or TST for TBI testing, emphasizing that IGRA should not replace TST in lower-middle– and low-income countries.

### Advancements in TBI diagnostics

The advancements in TBI diagnostics have been essential in improving the accuracy and reliability of tuberculosis infection detection. The absence of a definitive gold standard for diagnosing TBI presents significant challenges in outcome classification. However, recent progress in the development of novel TBI diagnostics has shown promise. One notable advancement is the introduction of the Cy-Tb test, an antigen-based skin test approved by India's regulatory authority for use in individuals aged over 1 year. This test uses M antigens specific to TB, similar to those used in IGRAs. The Cy-Tb test maintains sensitivity comparable to that of the traditional TST while addressing cross-reactivity issues associated with BCG vaccination, which can affect TST results. Compared with TST and IGRA, the Cy-Tb test offers unique advantages for programmatic use in resource-limited settings. Unlike IGRAs, which require laboratory infrastructure and are more expensive, the Cy-Tb test is simpler to administer and interpret. In addition, it avoids some of the logistical challenges associated with TST, such as the need for multiple visits and cold chain maintenance. Its potential for broader applicability in field settings makes it a promising option for scaling up TBI diagnosis in India.

In addition, a new iteration of the QuantiFERON test, known as the fourth-generation QuantiFERON-TB Gold Plus assay, has been globally introduced, contains an additional antigen tube (TB2), stimulating clusters of differentiation (CD)4+ and CD8+ T cells [21]. This updated version incorporates new antigens aimed at enhancing test sensitivity, particularly in immunocompromised populations, such as individuals living with HIV. However, initial independent evaluations have suggested only a modest increase in sensitivity for this updated test.

### Barriers influencing the availability and accessibility of TBI diagnosis

TBI testing is desirable whenever feasible to identify persons at the highest risk for developing active TB. However, it is not required in PLHIV or in household contacts aged <5 years. In HIV-negative household contacts aged 5 years and older and in other risk groups, TBI tests are recommended, but their unavailability should not be a barrier to treating people who were judged to be at higher risk [4].

IGRA testing is costlier than TST and requires appropriate laboratory services. Operational difficulties are present in low- and middle-income countries within resource-constrained settings. With easy availability and low cost, TST can be performed in the field, but it requires a cold chain; two health care visits; and training in intradermal injection, reading, and interpretation. One other practical advantage of IGRAs over TST is that they are not susceptible to a “booster response,” which makes a two-step approach necessary in situations where the reactivity to TST has waned since infection [20].

Despite all advancements, there remains variability in the use of TBI diagnostic tests worldwide. As reported, 116 countries, including India, primarily use either TST or IGRA for TBI screening, indicating their widespread adoption. In contrast, a smaller number of countries, eight in total, have adopted antigen-based skin tests for TBI diagnosis. Notably, 36 countries reported no use of any TBI diagnostic tests, with the majority of these countries located in the African region [1]. The introduction of novel diagnostic tools, such as the Cy-Tb test, represents a significant step forward in enhancing TBI detection under programmatic conditions, aligning with India's NTEP recommendations [22].

## India's policy expansion and response to tuberculosis

Seven of the eight nations of Southeast Asia (SEA) regions in the situational analysis—Bangladesh, India, Indonesia, Myanmar, Nepal, Thailand, and Timor-Leste—revised their National Tuberculosis Programs (NTP) and TPT policies in 2020-2021. India's NTEP carefully examined and considered these guidelines, leading to the creation of its own guidelines for managing TPT in 2021 [4]. India has established routine TB surveillance systems for TPT recording and reporting in the Ni-kshay Portal. At the 2018 United Nations High-Level Meeting on TB, countries committed to providing TPT to 40 million individuals by 2022. India's specific target within this was to provide TPT to 6.03 million people, categorized by age and risk factors [23]. The United Nations High-Level Meeting in 2023 revised global targets, now aiming to reach 45 million individuals over the 5-year period from 2023 to 2027. This expanded focus underscores the continued global commitment to scale-up TPT as a key strategy in TB elimination [24].

### District-specific planning

Recognizing the heterogeneity in TB burden across different regions, India emphasizes decentralized planning efforts. District-level TB control units conduct needs assessments and develop context-specific plans to ensure efficient resource allocation and program tailoring to meet local needs. The NTEP also conducted extensive training sessions for healthcare workers at national and regional levels to ensure they are equipped to implement the programmatic management of TPT guidelines effectively [5]. However, the ambitious 2025 TB elimination targets now appear difficult, given the systemic challenges and delays caused by the COVID-19 pandemic. The pandemic has significantly disrupted case detection, treatment adherence, and TPT coverage due to interruptions in healthcare services. These compounded factors necessitate a strategic intensification of efforts in the coming years, aiming to better align with the global 2030 TB elimination goals.

## India's position in scale-up of tuberculosis preventive treatment: analysis of Global Tuberculosis Report 2023

### Estimated number of eligible contacts for tuberculosis preventive treatment

In 2022, the WHO estimated that worldwide, 12.5 million household contacts, including 1.56 million children aged under 5 years, were eligible for TPT across 179 countries. For India, the estimation indicated the need for TPT in 3.6 million household contacts, including 0.36 million children aged under 5 years, representing 28.7% and 23.1% of the global estimation, respectively, as shown in Table 1.

**Table 1:** Estimated number of contacts eligible for TPT (Global and India), as per the Global TB Report 2023.

Variable	Global estimation (N)	India estimation (N, % of global estimation)
Estimated number of household contacts eligible for TPT	1,25,34,574	36,00,000 (28.7%)
Estimated number of children aged <5 years in contacts eligible for TPT	15,61,190	3,60,000 (23.1%)

TB, tuberculosis; TPT, TB prevention treatment.

### TBI screening in household contacts

In 2022, India reported 3.36 million household contacts, which was 93% of the total compared with global 71% of the total estimate. Among them, India screened 3.16 million household contacts, which was 94% compared with the global coverage of 84% of all enumerated household contacts, as shown in Table 2.

• Observations revealed a 7% and 6% gap in reporting and screening of household contacts, respectively, compared with a global total gap of 29% and 16%. Worldwide, 0.198 million (3%) TB cases were reported among those screened, with India reporting 14,175 (0.45%) cases. The wide variation among countries could be attributed to differences in coverage, diagnostic algorithms, and reporting completeness.
• India ranked 11th and 5th among 33 high-burden countries in the completeness of enumeration and screening of household contacts. However, India's reported TB detection rate of 0.45% after screening fell significantly short of the benchmark (∼4-5%), indicating to create a framework of strong monitoring for the quality of symptom screening and the availability of chest X-ray with AI-based computer-aided diagnosis with the WHO-approved rapid molecular diagnostics for TB diagnosis [25].

**Table 2:** Numbers of estimated, reported, and screened household contacts, newly diagnosed TB cases, and individuals provided with TPT across countries and India.

Variable	Total (Global) N (%)	Total (India) N (%)	Global rank of India among high-burden countries
Estimated number of household contacts of microbiologically confirmed TB cases	1,25,34,574	36,00,000	(28.7% of global estimation)
Reported number of household contacts (% of estimated)	89,53,459 (71%)	33,56,272 (93%)	11^th^
Screened number of household contacts (% of enumerated)	75,25,451 (84%)	31,61,022 (94%)	5^th^
New TB diagnosed from the screened number of household contacts (% of screened)	1,98,646 (3%)	14,175 (0.45%)	33^rd^
Number of household contacts provided TPT (% of estimated contacts)	19,49,054 (16%)	8,12,311 (22%)	-
Number of household contacts aged <5 years provided TPT (% of estimated contacts)	5,96,107 (38%)	1,68,665 (47%)	-
Increment in % of household contacts provided TPT in 2022 compared to 2018	∼4 times	∼9 times	-

TB, tuberculosis; TPT, TB prevention treatment.

### Progress in providing TPT to household contacts

India reported 0.81 million household contacts (including 0.17 million household contacts aged <5 years) initiated on TPT, as shown in Table 2, which is nine times from 2018. In 2021, India's TB program overcame policy barriers, facilitating monitoring and expansion of TPT among household contacts of all ages, crucial for reducing TB screening gaps and initiating TPT [26].

### Advancement toward predefined goals

India reported the initiation of TPT among 22% (13,09,584) of estimated household contacts, surpassing the global average of 16% (41,52,599).

### TPT completion rates

It was observed that 87 countries reported data on the outcome of TPT initiated in household contacts of persons with TB reported in 2022. The median TPT completion rate was 88%. India reported 70% TPT completion of those household contacts initiated on TPT in 2021 [1]. Only 6 months of daily isoniazid (6H) regimen was available in 2021 under India's NTEP, whereas the program strived to procure and introduce a shorter 3HP regimen. Lesser TPT completion with 6H than 3HP was observed in multiple studies. One of the reasons for the low TPT completion rate in India could be a longer 6H regimen. The reporting system of TPT under NI-KSHAY was also upgraded in 2021, and some of the data might be lost during data migration [27].

## Challenges in the implementation of TPT

In a regional study conducted under Project Akshya Plus (2021-2024), the delay was observed in the TB preventive care cascade. The median time between treatment initiation of index persons with pulmonary TB and (a) HHC screening was 31 days; (b) TPT initiation was 64 days [28]. By 2020, all 11 SEA nations have updated their TPT target groups and eligibility criteria and executed at least one shorter TPT regimen per the WHO guidelines. TPT scaling up in the SEA Region is constrained by scarce resources, provider and client knowledge and service delivery gaps, and rifapentine shortages for shorter TPT regimens [29].

### Health system-related challenges

Infrastructure and human resource scarcity pose significant obstacles, limiting the capacity to provide TPT services efficiently. Moreover, the availability of TPT drugs, along with procurement challenges, leads to interruptions in treatment continuity [7,28]. The lack of accessible diagnostic tools for identifying TBI further complicates TPT delivery. In addition, issues with data entry and system management hinder the effective monitoring and evaluation of TPT programs [30].

### Community-related challenges

The substantial population in need of TPT services poses logistical challenges in reaching and engaging all individuals. Moreover, TB-related stigma persists, leading to fear and discrimination, which hinder community cooperation and engagement. Refusal of contact tracing visits from healthcare workers is often driven by concerns about privacy and the stigma associated with TB. Limited awareness about TPT among the community further affects the uptake of services [30].

### Tuberculosis preventive treatment eligible population-related challenges

The prolonged duration of treatment regimens often leads to TPT non-adherence, affecting treatment outcomes. Moreover, resistance to isoniazid complicates TPT effectiveness, particularly, with monotherapy regimens. TB contacts or eligible population may also express reservations about initiating TPT, especially if they are asymptomatic or unaware of the benefits. Hesitancy from private health care providers further affects TPT coverage and access to services [30].

## Strategies and approaches to address present challenges

The next phase of tuberculosis elimination in India requires cost-effective interventions tailored to the diverse tuberculosis epidemiology. Continuous innovation, locally driven solutions, and rigorous program evaluation are essential.

### Addressing health system-related issues

To address these challenges, several solutions can be implemented, such as establishing a centralized procurement system for TPT drugs, which can ensure consistent availability and alleviate supply chain issues. Continuous capacity-building efforts, including training and support for healthcare institutions involved in TPT delivery, can enhance service delivery. In addition, the implementation of robust monitoring mechanisms is essential for tracking TPT data and addressing system issues promptly [29]. Collaboration among diverse stakeholders, including public and private sectors, national health authorities, Non-Government Organizations (NGO), researchers, and the pharmaceutical industry, is vital for successful implementation [31].

### Strengthening community engagement

Strengthened community engagement efforts are necessary to overcome these challenges. This can involve fostering community involvement through education, outreach, and the involvement of local champions. Educational campaigns aimed at raising awareness about TPT can help target misconceptions and reduce stigma. Using technology-driven communication tools and customized community outreach programs can enhance engagement and uptake of TPT services.

### Enhancing tuberculosis preventive treatment eligible population and provider support

A multifaceted approach is required to address these challenges requires. Introducing shorter-duration treatment options, such as the 3HP regimen, can improve TPT adherence and treatment completion rates. Capacity-building efforts, including training healthcare staff on TPT protocols and management of drug-resistant cases, can enhance service delivery and care among persons who are eligible for TPT. Engaging private health care providers through partnerships and incentive programs can expand access to TPT services and improve coverage. Establishing TPT eligible population and provider support schemes can foster collaboration and trust, encouraging eligible population to participate in TPT programs and adhere to treatment regimens [32].

## Conclusion

India's commitment to expanding TPT is evident through its strategic framework of policy expansion, political and administrative commitment, and notable progress in achieving TPT targets. Enhancing TPT in India requires continuous monitoring for real-time insights into the program's effectiveness, including quantitative assessments of TPT coverage and qualitative evaluations of person eligible for TPT's experiences, healthcare provider perspectives, and systemic bottlenecks. Strengthening TPT demands collaborative efforts from health authorities, communities, civil society, and the private sector.

## Declarations of competing interests

The authors they have no competing interests to declare.
